# Artificial Intelligence-Based Optimization of Pulmonary Drug Delivery Performance in Smart Inhaler Drug–Device Combination Systems

**DOI:** 10.3390/pharmaceutics18081026

**Published:** 2026-08-19

**Authors:** Harshada B. Pawar, Pawan Ganesh Nayak, Amatha Sreedevi, Ramya Ravi, Pradeep M. Muragundi

**Affiliations:** 1 Department of Pharmaceutical Regulatory Affairs and Management, Manipal College of Pharmaceutical Sciences, Manipal Academy of Higher Education, Manipal 576104, Karnataka, India; harshada.mcopsmpl2024@learner.manipal.edu (H.B.P.); amatha.mcopsmpl2022@learner.manipal.edu (A.S.); ramya.ravi@manipal.edu (R.R.); 2 Department of Pharmacology, Manipal College of Pharmaceutical Sciences, Manipal Academy of Higher Education, Manipal 576104, Karnataka, India; pawan.nayak@manipal.edu

**Keywords:** artificial intelligence, machine learning, data security, cybersecurity, inhalation systems

## Abstract

Advancements in pulmonary drug delivery have enabled effective treatment approaches for more severe disease conditions, such as chronic obstructive pulmonary diseases, asthma, cystic fibrosis, and other pulmonary disorders, via targeted, sustained, and immediate drug delivery routes with minimal systemic side effects. However, conventional delivery systems have many limitations, such as poor drug targeting, adherence, and deposition, which ultimately cause variations in drug profiles and therapeutic efficacy. Recent advances in artificial intelligence (AI) and machine learning (ML) have enabled the development of smart inhaler drug–device combination systems for personalized therapy using predictive formulation parameters, design variables, device performance, and inhalation pattern monitoring. Advanced AI techniques, such as artificial neural networks, deep learning, random forests, support vector machines, deep learning algorithms, and computational modeling, predict the mass median aerodynamic diameter (MMAD), fine-particle fraction (FPF), emitted dose, and regional lung deposition. Smart inhalation devices coupled with digital sensors and computing systems enable the real-time monitoring of inhalation profiles and adherence. Moreover, AI- and ML-enabled Quality by Design (QbD) and digital twin framework technologies enhance the optimization of manufacturing process parameters, consistency, robustness, and scale-up performance. Although several developments have been reported, there is still room for improvement in terms of data heterogeneity, algorithm transparency, interpretability, cybersecurity, regulations, and long-term clinical standardization. This review emphasizes the use of AI to improve the performance of pulmonary drug delivery through smart inhaler drug–device combination therapies, focusing on technological advancements, formulation optimizations, smart inhalers, regulatory issues, current limitations, and future perspectives of AI-based pulmonary drug delivery.

## 1. Introduction

Therapeutic pulmonary drug administration has become a major strategy for treating respiratory conditions such as asthma, chronic obstructive pulmonary disease (COPD), cystic fibrosis, pulmonary hypertension, and pulmonary infections, as it ensures the direct delivery of medication into the lungs, rapid onset of action, minimal side effects, and increased efficacy [1,2,3]. Many physiological benefits are associated with the pulmonary approach, including a large surface area of approximately 100 m^2^, high vascularization, and a thin alveolar layer, which facilitates drug absorption and deposition [3]. Inhalation devices, such as pressurized metered-dose inhalers (pMDIs), dry powder inhalers (DPIs), nebulizers, and soft-mist inhalers (SMIs), are among the most commonly used inhalation devices for treating respiratory disorders; however, the efficacy of this therapy is compromised by low deposition efficiency, erratic drug delivery, and patient variability [2,3,4].

The main problems associated with inhalation therapy include poor inhaler usage or compliance among patients with asthma and COPD. Many patients with asthma and COPD use inhalers improperly, which significantly affects the treatment results [5,6,7]. Conventional pMDIs are actuation- and inhalation-based, whereas DPIs are highly dependent on the rate and strength of inhalation, which can change with disease stage, age, and respiratory function [4,6,7]. Moreover, inhalation patterns and variations in the formation and size of aerosols may negatively affect lung deposition and increase the risk of drug absorption via the oral cavity [3]. These restrictions have encouraged researchers to develop modern pulmonary drug delivery devices that can control the drug delivery process and monitor patient inhalation.

The latest innovations in artificial intelligence (AI) and machine learning (ML) have revolutionized the development of smart inhalers and pulmonary drug delivery systems (PDDSs) [1,8]. AI-powered systems are increasingly being applied to formulation design, aerosol performance prediction, inhaler optimization, patient adherence monitoring, and personalized treatment [1,8,9]. Jiang et al. reviewed the increasing use of AI and ML in pulmonary drug delivery, including disease identification, computational aerosol science, formulation design, and digital therapeutic monitoring [1]. The application of AI in pharmaceutical research and the utilization of predictive models and data-driven processes have accelerated formulation screening, process parameter optimization, and inhalation behavior improvements with lower experimental workloads and development costs [1,10,11].

AI technologies, such as artificial neural networks, deep learning, random forests, and support vector machines, predict aerosol properties, enhance device efficiency, and simulate inhalation profiles based on patients [12,13,14]. These computational methods enable accurate the prediction of critical aerosol performance parameters, including MMAD, FPF, emitted dose, and regional lung deposition [1,15]. CFD and CFPD simulations, together with MI algorithms, predict how aerosols are transported and deposited during inhalations under varying conditions, facilitating the design of personalized inhalation devices [15,16].

Smart AI-enabled inhalers effectively monitor and manage inhalation. Chamaon et al. confirmed that an AI-enabled smart DPI (RS01X) is effective in measuring inhalation metrics, including inspiratory volume, peak inspiratory flow (PIF), and time to inhalation, in patients with asthma and COPD [17]. The AI-enabled smart inhaler demonstrated good agreement with external inhalation monitoring tools and provided real-time feedback on inhalation techniques and correct device use [17]. In addition, digital inhaler systems featuring flow rate sensors and technology can be used for continuous inhalation monitoring and the delivery of clinical data to healthcare professionals [18,19]. Mysovskikh et al. reviewed the latest developments in portable smart inhaler flow rate monitoring [18].

Other significant technological breakthroughs in AI-driven lung drug delivery include the design of a computational intelligence-based smart inhaler that can target aerosol delivery according to patients needs. Islam et al. created a smart inhaler trained using computational fluid dynamics-based MI (CFD-ML). This inhaler optimizes aerosol delivery to the small airways by customizing the operational parameters according to individual breathing patterns and aerosol characteristics [16]. This finding highlights the potential for enhancing targeted aerosol delivery with reduced deposition [16].

Similarly, AI supports QbD and process optimization for the manufacture of inhaled drugs. This refers to the use of AI-based pharmaceutical platforms to analyze large sets of data obtained from processes such as spray drying, mixing, particle design, and packaging to determine critical process variables and predict the quality of the end product [1,10]. AI technologies improve scale-up, manufacturing reproducibility, and process monitoring [1,8]. Moreover, regulatory organizations, such as the Food and Drug Administration (FDA) in the U.S., have acknowledged the importance of AI and MI technologies in medical devices and pharmaceutical development.

However, several challenges remain in the application of AI in drug delivery via pulmonary devices. The first problem is that AI algorithms use small or heterogeneous datasets, which could influence the generalizability of the models among different patients and inhalers [1,8]. Other issues that should be mentioned are data security, algorithm transparency and interpretability, cybersecurity, and standardization [8,9,20]. Clinical studies and post-marketing surveillance systems are required to assess the efficacy, safety, and performance of these intelligent inhalers.

### Novelty and Scope of the Present Review

The field of drug delivery via inhalation has evolved considerably with the integration of artificial intelligence (AI) and machine learning (ML). A recent comprehensive review has highlighted the applications of these technologies in formulation optimization, aerosol performance prediction, computational fluid dynamics (CFD)-based modeling, smart inhalers, digital inhalation devices, and AI-assisted drug development. Furthermore, the review discussed the role of AI and ML in the design, optimization, and evaluation of inhalation drug delivery systems and inhalation devices [1]. However, this review primarily focuses on individual domains of AI-enabled pulmonary drug delivery and does not comprehensively integrate pharmaceutical formulation development, smart inhaler drug–device combination systems, digital twin technology, AI-enabled quality by design (QbD), manufacturing process optimization, regulatory science, and translational implementation within a single review.

This review addresses this need by providing a comprehensive interdisciplinary perspective on AI-assisted pulmonary drug delivery across the entire product lifecycle. In contrast to previous reviews, the current study combines AI-guided formulation development, prediction of the key performance characteristics of aerosols, computational fluid dynamics, smart inhaler drug–device combination systems, digital twin technologies, AI-driven Quality by Design (QbD), optimization processes of manufacturing, and regulatory aspects in a single framework. Furthermore, rather than merely summarizing published studies, this review critically evaluates the strengths, limitations, clinical applicability, and translational potential of current AI-driven approaches in the field. Particular emphasis is placed on challenges related to data quality, model interpretability, external validation, cybersecurity, regulatory acceptance, and real-world implementation, while identifying existing knowledge gaps and future research priorities.

By integrating evidence from pharmaceutical sciences, biomedical engineering, computational modeling, digital health technologies, and regulatory science, this review provides a holistic perspective on the evolving role of AI in pulmonary drug delivery and smart inhaler drug–device combination systems. The comprehensive synthesis presented in this review is intended to support researchers, clinicians, pharmaceutical scientists, engineers, and regulatory professionals in accelerating the safe, effective, and clinically meaningful translation of AI-enabled pulmonary drug delivery systems into clinical practice.

## 2. Literature Search Approach

A narrative literature search was conducted to identify studies describing AI and ML applications in pulmonary drug delivery and smart inhaler drug–device combination systems using various electronic databases, including PubMed, Scopus, Web of Science, and Google Scholar. The search also considered regulatory documents provided by the U.S. Food and Drug Administration (FDA) and other relevant sources.

The keywords “artificial intelligence,” “machine learning,” “pulmonary drug delivery,” “smart inhalers,” “digital inhalers,” “computational fluid dynamics,” “digital twins,” “quality by design,” “aerosol performance,” AND and OR “inhalation drug delivery,” were used in combination with the Boolean operators AND and OR. This study also considered peer-reviewed original and review articles published in English. Further articles were identified at a later stage by screening the reference lists of selected articles.

The identified literature was critically reviewed and synthesized in a narrative form to present the available information regarding the features, applications, difficulties, regulatory issues, and future possibilities of AI-enabled pulmonary drug delivery and smart inhaler drug–device combinations.

## 3. Essential Aspects of Pulmonary Drug Delivery

Pulmonary drug delivery can be defined as the inhalation of medicines directly into the lungs in the form of aerosols, dry powders, or nebulized liquid particles to exert local or systemic therapeutic effects. [1,21]. The respiratory system has several physiologically advantageous features for drug delivery, including an enormous absorptive area with a surface area of approximately 100 square meters, high vascularization, low enzymatic activity, and thinness of the alveolar lining membrane (approximately 0.1–0.2 micrometers), allowing drugs to be rapidly absorbed by the body [21,22]. Pulmonary drug delivery systems have proven to be highly effective in treating a wide range of respiratory conditions, such as asthma, chronic obstructive pulmonary disease (COPD), cystic fibrosis, pulmonary hypertension, and respiratory infections, and are increasingly utilized for the systemic delivery of peptides and proteins [22,23].

### 3.1. Classification of Inhalation Drug Delivery Systems

**Figure 1:**
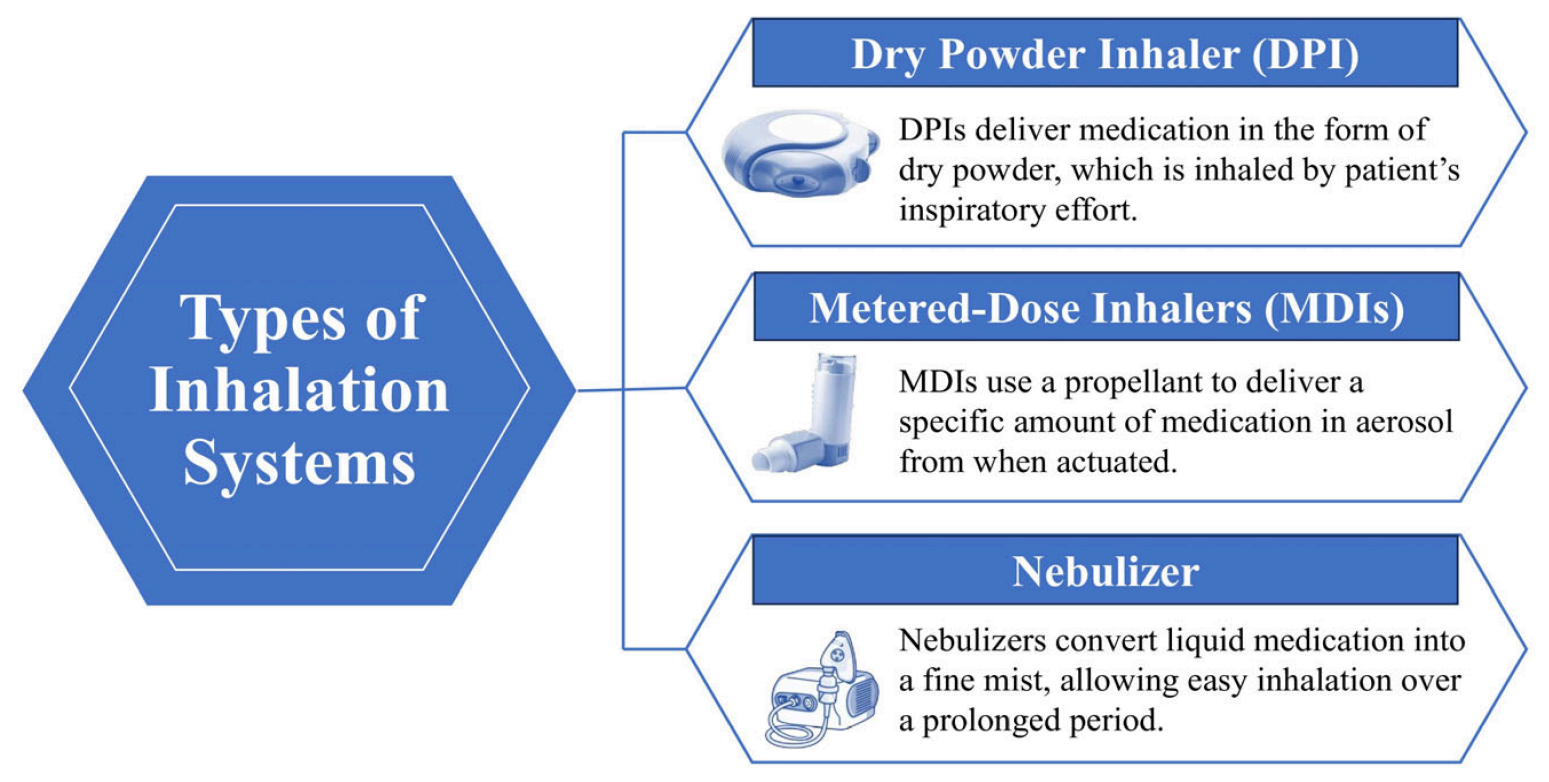
Types of inhalation systems.

Three major inhalation drug delivery systems used for pulmonary drug delivery: dry powder inhalers (DPIs), metered-dose inhalers (MDIs), and nebulizers [3,24] (Figure 1). These devices are characterized by differences in the mechanisms of aerosol generation, patient inspiratory effort, particle size, and manner of deposition in the respiratory tract.

#### 3.1.1. Dry Powder Inhalers (DPIs)

DPIs administer aerosolized drugs as dried powders suspended in the inspiratory airflow [25,26]. Many commercial DPI formulations consist of micronized drug particles mixed with lactose particles to increase the efficiency of powder suspension [26]. Inhaled dry powders require turbulent energy generated by the inspiratory flow rate and resistance of the device for efficient aerosolization [3,27]. The typical inspiratory flow rates of DPIs vary from 30 to 90 L/min [3].

Particle size is an important aerodynamic property of inhaled powders that determines their fate in the lung. The particles used for inhalation are usually designed to have aerodynamic sizes in the range of 1–5 µm to enable effective deposition in the lower airways [3,21]. Improved DPI designs include the use of aerodynamically enhanced flow channels, turbulence chambers, and mouthpiece modifications to enhance aerosol dispersion and increase fine-particle fraction (FPF) [3,25]. Several studies demonstrate that optimized DPI geometries can significantly improve aerosolization efficiency and lung deposition performance compared to conventional inhaler designs [3]. Commonly available DPIs include Turbuhaler^®^, Diskus^®^ (Accuhaler^®^), and Breezhaler^®^, which are extensively used to deliver bronchodilators and corticosteroids for asthma and COPD treatment.

#### 3.1.2. Metered-Dose Inhalers (MDIs)

Aerosols from metered-dose inhalers are created by the ejection of a liquid dose using HFA propellants through a pressurized actuator. The rapid evaporation of the propellant during actuation creates droplets that become respirable particles capable of lung deposition. Aerosols created by conventional MDIs usually have MMAD values between 2 and 5 µm, making them ideal for deposition in the bronchi and small airways [3].

The need to coordinate inhalation with actuation is a key disadvantage of MDIs, particularly in pediatric and elderly patients [4]. Miscoordination leads to poor lung deposition and oropharyngeal loss of the drug [23,28]. Spacers and valved holding chambers are often used to increase deposition in the lungs and decrease mouth and throat deposition [3]. Compared to conventional single-orifice devices, modified nozzles with multiple-orifice actuators have been proposed to provide better aerosol dispersion and higher FPF values [29]. Commercially available pMDIs, such as Ventolin^®^ Evohaler (salbutamol) and Symbicort^®^ (budesonide/formoterol), are widely used to manage asthma and chronic obstructive pulmonary disease (COPD).

#### 3.1.3. Nebulizers

Nebulizers transform liquids into aerosols using air compression, ultrasonication, or vibrating-mesh mechanisms [3]. In contrast to DPIs and MDIs, nebulizers do not require coordination from patients and are thus appropriate for use in pediatrics, severely ill patients, and patients with impaired breathing function [3,22]. Aerosols formed by nebulizers have MMAD sizes of approximately 2–5 µm, depending on the viscosity of the formulations and nebulizer mechanisms [3].

Vibrating-mesh and sophisticated jet nebulizers can produce aerosols with MMAD values ranging from 1.5 to 3 µm, which helps with the deposition of drugs more effectively in the peripheral lungs [30]. Clinical trials on patients with COPD and cystic fibrosis have shown significant improvements in drug deposition in the deep lung in response to nebulized aerosols with MMADs below 2 µm compared to other particles [30]. Moreover, modern nebulizer systems can minimize the volume of leftover formulations and provide consistent emitted doses across different inhalations [3]. Commercial nebulizer systems, such as the PARI LC Sprint^®^ Nebulizer and Philips Respironics InnoSpire Essence^®^, are widely used to deliver bronchodilators, corticosteroids, antibiotics, and mucolytic agents to patients with asthma, COPD, and other respiratory disorders.

### 3.2. Key Performance Parameters

The performance of pulmonary drug delivery systems is analyzed using different aerosol parameters, including particle properties that influence aerosol deposition efficiency [1,3].

#### 3.2.1. Mass Median Aerodynamic Diameter (MMAD)

The MMAD represents the diameter at which equal parts of the aerosol mass are divided into larger (>d) and smaller (<d) particles [3]. The MMAD is a key parameter that influences the deposition process in the pulmonary system [21]. Particles exceeding 5 µm in diameter can deposit mainly through inertial impaction in the oropharyngeal region and upper airways, whereas particles with a diameter of less than 1 µm may be exhaled without deposition [21,30]. The optimal range of the aerodynamic particle diameter for deposition in the deep lungs is usually within 1–5 µm, whereas particles from 1 to 3 µm are especially suitable for deposition in the alveoli and peripheral bronchi [3,21]. Small aerosols with MMADs under 2 µm are widely used to treat asthma and COPD because of their ability to penetrate small airways and increase deposition in diseased lungs [24]. MMAD can be measured using cascade impactors, such as Andersen or next-generation impactors, operated under certain airflow conditions [31].

#### 3.2.2. Fine-Particle Fraction (FPF)

The FPF is defined as the percentage of aerosol particles with an aerodynamic diameter of ≤5 μm that can enter the lower airways [3]. The FPF value is reported as a percentage of the emitted dose and can serve as an index of aerosol quality and effectiveness for lung deposition [32]. Enhanced DPI and advanced MDI systems have been reported to improve aerosol dispersion and achieve higher FPF values than conventional inhalers [3,29]. The factors that influence FPF include particle characteristics, inhalation flow rate, powder cohesion, inhaler resistance, and actuator design [3,25]. Because the FPF alone does not completely describe aerosol distribution, it is sometimes evaluated alongside the geometric standard deviation (GSD) and aerosol distribution by cascade impact to predict in vivo aerosol behavior [32,33].

#### 3.2.3. Emitted Dose

The emitted dose is defined as the quantity of drug emitted from an inhaler during actuation or inhalation and can be determined using filters, impactors, or sampling devices positioned downstream of the inhaler mouthpiece [3]. The emitted dose of MDIs is usually expressed in micrograms per actuation, whereas that of DPIs is usually provided as a percentage of the labeled dose collected after inhalation [3]. Regulatory guidelines require that the emitted dose uniformity remains within predefined acceptance limits relative to the labeled claim to ensure consistent dose delivery [34]. The emitted dose depends on several factors, including the formulation ingredients, precision of the metering valve, powder fill consistency, inhalation flow rate, and device design [1]. The emitted dose is an important criterion used in optimization studies of pulmonary drug formulations assisted by AI [1,35].

#### 3.2.4. Deposition Efficiency

Deposition efficiency is a measure of the amount of aerosols that are actually deposited into the lungs from the inhalation process rather than being wasted through the use of inhalation devices and the anatomical structures involved in breath delivery (e.g., mouth, pharynx, and exhalation) [21]. Historically, the amount of aerosol deposited in the lungs when an inhaler was used averaged only 10–20% of the entire amount delivered to the mouth/throat/lungs [4,21]. The amount of aerosol deposited in the lungs from the inhaler also depends on the size (diameter) of the aerosol particle, as well as many other factors, such as the rate of airflow through the inhaler during inhalation, the configuration of the person’s airways, and various medical conditions affecting the person’s ability/usage of inhalers and breathing [21,30]. Modern developments in computational fluid dynamics (CFD) and computational fluid particle dynamics (CFPD) models have provided new ways to simulate the deposition process in individual patient airways [16,32]. AI-based smart inhalers integrate airflow sensors, deposition estimation algorithms, and real-time inhalation measurements to increase aerosol delivery efficiency based on individual respiratory characteristics [1,16,17].

## 4. Drug–Device Combinations in Inhalation Systems

Inhalation drug delivery systems use different forms of inhalers (DPIs, MDIs, and soft-mist inhalers) or nebulizer–drug systems. By using various devices with established drug formulations, the combination of drugs and devices enhances therapeutic benefits to patients and can be considered a combination product. The FDA defines a combination product as two or more regulated components that are combined physically or functionally to deliver therapeutic benefits [36]. The inhaled drug in each type of inhalation system determines the aerosolization of the drug from the device (i.e., the emitted dose uniformity, aerodynamic particle size distribution, and regional lung deposition) [37,38]. Consequently, the CDRH guidance for MDI and DPI products requires a combined evaluation of device design, formulation compatibility, emitted dose uniformity, aerodynamic particle size distribution, and reproducibility during development and manufacturing processes [37].

The performance of inhalation combination products depends largely on the performance of the device used, specifically how well the device builds up and releases pressure during the inhalation process. With DPIs, the patient-generated inspiratory airflow supplies the energy needed to disperse the powder into small particles and create an aerosol. The resistance produced by the inhaler during the inhalation process is determined by the construction features that control the path of the aspiration airflow, baffles (slats that break up ambient air), mouthpiece geometry, and inlet structures that control the turbulence generated by the gas as it enters the inhaler. For DPIs to provide clinically relevant flow rates to the patient from approximately 30–90 L/min, on the basis of the device used and the patient’s natural respiratory function, inhalers that have high resistance provide greater turbulence that may result in the better dispersal of cohesive formulation powders than low-resistance inhalers that offer greater ease in the passage of gas, though the device will often require stronger inhalation to aerosolize the formulation adequately for the patient to use. De Boer et al. used the literature findings of inhaler testing based on design and internal aerodynamics to illustrate how the FPF and emitted dose of an inhaler can be influenced by both the geometrical design of the inhaler and the amount of resistance to flow through the inhaler [39]. Puri et al. evaluated inhalers using computational fluid dynamics (CFD) and reported that the performance of capsule DPIs is strongly affected by the forces holding the formulation to the capsule and the inhaler’s aerodynamic performance [40].

The aerosols produced by different types of inhalers vary greatly in terms of their formation, which affects the way they are formed. One of these effects is the size of the aerosol produced (measured by its MMAD), which affects the FPF of the aerosol (3). When a DPI is used, the shear and turbulent forces produced by the inspiratory airflow cause the separation of the micronized active pharmaceutical ingredient (“API”) particles from the lactose carrier particles. (26,39,41). The dispersion and reproducibility of the dose depend on the characteristics of the powder filling the DPI, such as the particle size, morphology, surface roughness, and electrostatic properties [41]. In contrast, when an aerosol is produced by an MDI, it is created through the rapid release of the hydrofluoroalkane (“HFA”) aerosol propellant through the metering valve and actuator nozzle of the inhaler [3]. During HFA evaporation, aerosols created are normally within the MMAD range of 2–5 µm in size for delivery to the lungs [3]. Newman and Busse reported that the shape of the actuator, size of the nozzle, and shape of the plume significantly affected the velocity of the aerosol, as well as the amount of aerosol that will deposit in the upper airway during the use of MDI [42]. Aerosols may also be created using a nebulizer that uses compressed air, ultrasonic vibration, or a vibrating-mesh system, where the characteristics of the aerosol are influenced by the dimensions of the nozzle, the viscosity of the liquid used, and the operating pressure [3]. Inhalation products (aerosols) with identical formulation characteristics provide different deposition profiles depending on the device used for dispensing inhalation (the device). Appropriate formulation device co-development is required to achieve optimal therapeutic benefits [3,37].

The compatibility of the formulation and delivery device is a key aspect in the development of inhalation combination products. In carrier-based DPIs, the use of a carrier material (e.g., lactose) with a specific particle size (between 10 and 220 µm) is necessary to increase the flow of the powder and aid in the detachment of the micronized drug during inhalation [41]. The airflow architecture of the delivery device should create sufficient turbulence to adequately disperse the powder while minimizing the retention of the powder in capsules or dead space within the device. Research conducted by Rawal et al. revealed that the morphology of the carrier and the energetics of its surface significantly affect the ability of the active pharmaceutical ingredient (API) to detach from the carrier and the FPF of DPI formulations [41]. With respect to MDIs, formulation-related factors such as the surfactant (i.e., stabilizing material) concentration, propellant combination, and API loading interact with the actuators to affect droplet evaporation, plume angle, and mouth–throat deposition. Therefore, regulatory agencies require that both formulation and device performance be evaluated together throughout the production and distribution of inhalation combination products [36,37,38].

Variability in medication doses is a significant barrier to inhalation therapy and can be caused by several factors, either from the devices or the patients [43,44]. In one study, Newman et al. reported that while some multidose DPIs had greater in vitro variability in terms of the dose of medication, metered-dose inhalers had greater in vivo variability because of the poor coordination of actuation and inhalation [43]. Additionally, Munzel et al. reported that fine-particle dose variability and budesonide deposition within the lungs varied among multidose DPIs based on an inspiratory flow range of 40–100 L/min [44]. Incorrect handling of inhalers, inadequate inspiratory flow, and poor inhalation technique drastically decrease the deposition of medication into the lungs, thereby decreasing the effectiveness of the medicaytion [5]. A systematic review by Chrystyn et al. revealed that inhaler handling errors were common among patients with asthma and chronic obstructive pulmonary disease (COPD) and were related to poor disease control and an increased risk of exacerbations [5].

Digital inhalers integrate intelligent digital drug devices that monitor inspiratory flow rate, timing, orientation, and adherence in real time [17,18]. Chamaon et al. validated an AI-based smart DPI (RS01X) for measuring inspiratory parameters in patients with asthma and COPD with real-time feedback on the inhalation technique [17]. Mysovskikh et al. demonstrated that portable digital inhalers with integrated flow rate sensors can continuously monitor patient inhalation patterns and enable consistent dosing [18]. Zhang et al. designed an MI inhaler prototype based on computational fluid-particle dynamics simulations of aerosol delivery to adapt aerosol release based on an individual’s airways and breathing patterns [16]. Collectively, these advances demonstrate that the transition from passive inhalers to intelligent, AI-assisted inhalers may enable a more personalized therapeutic experience through more effective pulmonary delivery methods [16,17,18].

AI and ML optimize the development of pulmonary drug delivery formulations using methods such as DPIs, nanoparticle delivery systems, and spray-dried inhalable [36,37,38,45]. Traditional development relies on trial-and-error methods and the large design of experiment (DOE) matrices, which are typically time-consuming and create an additional resource drain [36]. In contrast, AI-driven methods/models evaluate large multidimensional datasets that include excipient ratios, particle morphology and size, aerodynamic performance, and process variables to enable the rapid identification of an appropriate formulation to achieve the desired MMAD, emitted dosage (ED), and FPF [36,37]. The characteristics, advantages, limitations, and pulmonary drug delivery applications of the major AI algorithms are summarized in Table 1.

## 5. Role of AI in Optimization

### 5.1. Role of AI in the Development of Formulations

Recent studies on lactose-containing DPI formulations have revealed that ML-guided optimization of the API-to-excipient ratios and carrier particle size markedly improved aerosolization [1,41]. Jiang et al. reported that AI-assisted QbD frameworks reduce the experimental workload and accelerate the optimization of pulmonary formulations while maintaining target aerosol performance parameters such as FPF and emitted dose [1]. Specifically, Random Forest (RF), Support Vector Machine (SVM), and gradient boosting ML algorithms were used to establish the optimal lactose carrier fraction and cohesive forces required to facilitate optimal powder dispersion [1]. Similar observations were made in thin-film freezing studies that employed ML-based methods to improve particle engineering and aerosolization efficiency while shortening the time required for the developmental processes [51]. As summarized in Table 2, AI applications in pulmonary drug delivery include formulation optimization, CFD-assisted modeling, smart inhalers, AI-QbD systems, and imaging analysis.

The development of MI systems has led to significant advancements in particle morphology and surface analyses [47,63]. Jeong et al. used scanning electron microscopy images of surface characteristics to create MI algorithms that predicted the aerodynamic behavior of the arformoterol–lactose formulations [47]. The aerodynamic behavior of each formulation was predicted by 13 features from scanning electron microscope images, such as kurtosis (Rku), skewness (Rsk), and asperity distribution, and using multilayer perceptrons (MLPs) and convolutional neural networks (CNNs [47]. MLP models combined with random forest (RF)-based feature selection demonstrated strong predictive performance for emitted dose (ED) and delivered dose (DD), whereas CNN models trained on SEM images showed better prediction performance for FPF and fine-particle dose (FPD) [47]. In general, the greater surface roughness and asymmetrical asperities associated with particles provided higher levels of deagglomeration, resulting in increased FPFs of the powders, despite a slight decrease in the emitted dose [47]. These findings suggest that AI-based morphological data supports the rational development of inhalable formulations.

The optimization of spray drying (SD) and milling (M) processes for pulmonary drug products is another important application of AI. SD generates inhalable particles with predetermined physical properties, including density, aerodynamic diameter, and moisture content [52]. Several critical process variables (e.g., inlet temperature, atomization pressure, feed concentration, and drying airflow) can be altered by adjusting the process parameters, which subsequently influence the physical and chemical characteristics of the final particles [52]. Digital-twin-based frameworks that use computational fluid dynamics (CFD) based on either mechanistic models or AI algorithms to predict particle size distributions and residual moisture have been shown to significantly outperform traditional mechanistic-based predictions in terms of particle size distributions and residual moisture prediction, thus demonstrating significant improvements in prediction accuracy for both pharmaceutical spray drying and milling processes [49]. AI-assisted and hybrid modeling approaches have shown improved predictive capabilities for physicochemical properties and process optimization in pulmonary particle engineering, contributing to improved scale-up performance and batch-to-batch reproducibility [49,52].

### 5.2. Role of AI in the Prediction of Aerosol Performance

The prediction of aerosol performance parameters, such as the FPF, MMAD, deposition efficiency, and airflow performance relationships [1,15], was substantially accelerated by AI. Conventional cascade-impactor techniques are also available for characterizing aerosol performance; however, the process is slow, labor-intensive, and requires multiple experiments [15]. AI-based surrogate models predict the aerodynamic performance of aerosols from formulation and device parameters [1].

Initial work with neural networks demonstrated that artificial neural networks (ANNs) accurately predict FPF for the DPI-based delivery of salmeterol and salbutamol using various input variables (e.g., drug load, carrier-fine content, and device resistance) [46]. Using a 9-4-1 neural network configuration, a high level of accuracy was achieved in predicting the FPF for the tested devices (i.e., R^2^ ≥ 0.92), indicating a strong nonlinear relationship between the formulation input variables and aerosol performance [46]. These models enable the virtual screening of DPI formulations, thereby reducing the reliance on repetitive AI, ML, computational fluid dynamics (CFD), and imaging technologies, which represent exciting avenues for optimizing pulmonary drug delivery systems. These technologies can be used to predict how aerosols travel through the air to the lungs, how aerosols are deposited inside the lungs when inhaled, and how inhalers perform in terms of delivering drugs efficiently to the patients. Thus, these technologies have greatly improved the design and development of both orally inhaled predictive modeling and intelligent inhalers [1,53].

Advanced imaging technology enabled researchers to better understand the retention time of nanoparticles in the lungs and the rate at which they are removed and cleared from the lungs following deposition. For example, LungVis 1.0 is a three-dimensional imaging technology that was used to examine how nanoparticles are distributed in the lungs and how quickly they are cleared from the lungs by macrophages in an animal model of lung disease. LungVis 1.0 enables detailed the spatial visualization of nanoparticle distribution and macrophage migration in murine lungs, supporting an improved understanding of pulmonary nanoparticle retention and clearance mechanisms [62]. These new technologies provide valuable information on both how and where to deliver nanoparticles into the lungs and how long nanoparticles will remain in the lungs following their airway and alveolar deposition.

AI plays a significant role in improving the understanding of airflow–performance relationships with inhalation apparatuses [56]. AI-based inhalation monitoring systems with integrated sensors can accurately classify inhalation techniques and airflow patterns with high precision [56]. AI-based inhalation monitoring systems have demonstrated high classification accuracy for inhalation flow profiles and inhaler technique assessments [56]. These systems identify inhalation errors in real-time and facilitate customized training with inhalers.

Different AI algorithms, such as artificial neural networks (ANNs), random forest (RF), support vector machine (SVM), gradient boosting, and deep learning, have shown suitable levels of performance in pulmonary drug delivery applications, although no single AI algorithm can be used as the optimal algorithm for all applications [1,35,46,47,48,50]. ANN and deep learning algorithms have high predictive power for complex nonlinear relationships and the prediction of aerosol performance; however, they usually require enormous, perfectly organized datasets and can be difficult to interpret because of their opacity [46,47]. Moreover, in contrast to the RF algorithm, which has high protection against overfitting as well as a reasonable possibility of identifying important predictive factors, the SVM algorithm shows good results based on relatively small datasets but has limitations in scalability for larger datasets [1,47,48]. Therefore, it is necessary to select the appropriate AI model based on the available data, complexity of calculations, and required level of interpretability. Additionally, it is crucial to validate the results of model prediction on different datasets as well as under clinical and manufacturing conditions [1,48,50].

### 5.3. Role of AI in Smart Inhaler Systems

**Figure 2:**
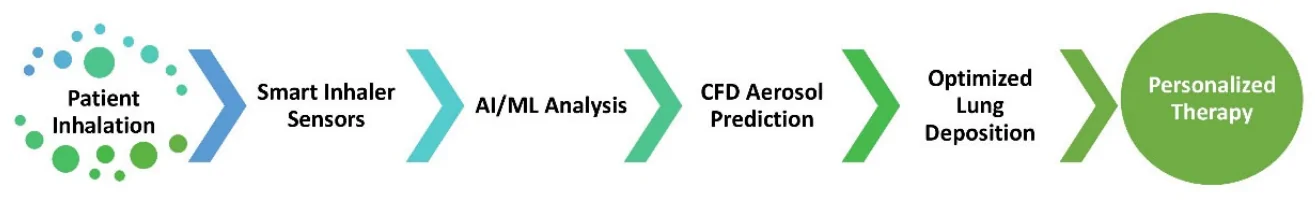
Workflow of the AI-enabled smart pulmonary drug delivery system.

The invention of AI-driven smart inhalers have transformed the traditional inhalation drug–device combination systems to intelligent digital therapeutic platforms [1,17]. Modern smart inhalers use flow sensors, accelerometers, microphones, and wireless communication technology to continuously monitor the inhalation profile, medication adherence, and user technique errors. Real-time MI algorithms hosted on the cloud analyze these data and detect errors within each event, including inadequate inspiratory flow, early termination of inhalation, poor coordination of inhaler use, and missed doses [17]. The general workflow of an AI-enabled smart pulmonary drug delivery system is illustrated in Figure 2.

The results of clinical validation studies indicate that AI-assisted smart inhalers help improve both the quality and adherence to inhalation therapy by patients [17]. Chamaon et al. validated the RS01X AI-assisted DPI in patients with asthma and COPD and reported a strong agreement between inhaler-recorded parameters and an external reference system, with intraclass correlation coefficients for inspiratory volume and peak inspiratory flow exceeding 0.95 [17]. The system also demonstrated the potential for real-time inhalation monitoring and feedback to support inhaler technique assessment and adherence [17].

Research has shown that smart inhalers utilizing AI and computer fluid dynamics (CFD) may help enhance the delivery of drugs into the lungs by monitoring patient actions while taking their medication, such as the speed of inhalation, duration of inhalation, and how they hold their device positioning while taking a puff [17,54,56]. Smart inhaler systems with MI can provide real-time information to patients regarding their inhalation techniques to support individualized respiratory therapy [17,56]. CFD-assisted smart inhaler prototypes have demonstrated reduced oropharyngeal deposition, whereas AI-based inhalation monitoring systems have shown high accuracy in predicting inspiratory flow parameters [17,54,56].

## 6. Role of AI in Quality and Process Optimization

The quality-by-design (QbD) framework for pulmonary drug delivery (inhalation) systems continues to employ layers of AI and ML in traditional QbD frameworks to optimize inhaled drug delivery systems, specifically through the use of drug delivery device combinations such as DPIs and MDIs [1,48,50]. The traditional implementation of QbD frameworks is based on trial-and-error methods for identifying critical material attributes (CMAs) and/or critical process parameters (CPPs) that affect the emitted dose, FPF, and the aerodynamic particle size distribution of inhaled products. This requires more experimental screening than traditional QbD methods until the CMA and/or CPP is identified [1]. AI/ML approaches are widely applied using supervised ML algorithms, such as random forest (RF) algorithms, artificial neural networks (ANNs), and gradient boosting models, to assist in the identification of critical quality attributes using limited clinical and experimental data [1].

As reported by Jiang et al., AI/ML has been applied to pulmonary drug delivery system development for virtual screening, formulation design, and the in silico optimization of inhaled formulations. In addition, AI-based predictive models have been used to estimate aerosol performance parameters, including fine-particle fraction (FPF), thereby facilitating the optimization of dry powder inhaler formulations [1,46,47]. AI-QbD approaches support the provision of a certified method for establishing predictable operating ranges (predictive operating windows) and using a risk-based control method (as opposed to a control method based on quality) in the processes of filling inhalers with the drug, sealing blisters containing the drug, and assembling the inhalers [1,48]. Additionally, according to Patel et al. (2020), ML-based QbD approaches improve the robustness of pharmaceutical manufacturing processes through the early identification of high-risk factors and real-time process improvement using ML algorithms [48].

DPI manufacturing processes, such as jet milling, spray drying, blending, and capsule filling, greatly benefit from AI-driven process optimization [50]. Emerging digital twin frameworks integrate empirical process data, computational modeling, and real-time sensor information to optimize manufacturing parameters, such as milling pressure, blending time, feed rate, and fill depth. AI-assisted process control approaches demonstrated improved process consistency, emitted dose reproducibility, and the maintenance of acceptable fine-particle fraction (FPF) performance during the manufacturing of inhalation products. Consequently, there is a high degree of consistency in pulmonary drug delivery performance [50]. AI-assisted multi-scale simulations and predictive modeling approaches may help improve manufacturing reproducibility and process robustness in DPI production [50].

AI-enabled predictive models improve the ability to predict scale-up performance using AI-supported multi-scale simulation frameworks. Nguyen et al. and Remmelgas et al. were among the earliest groups to quantitatively evaluate and compare DPI performance at different scales using discrete element modeling (DEM) and computational fluid dynamics (CFD) in combination with MI surrogate models. Their AI-based multi-scale simulations indicated that the MMAD can shift by approximately 0.3–0.5 microns and that the FPF can decrease by approximately 5–10 percentage points as manufacturers transition from laboratory-size operations to commercial-size operations, suggesting that it is critical to use AI-based predictions for scaling up to develop reliable pulmonary products [50]. Such predictive models enable proactive process controls prior to commercial-scale manufacturing, ultimately improving the efficiency, reproducibility, and regulatory robustness of smart inhaler drug/device combination systems.

## 7. Challenges and Limitations of AI-Enabled Pulmonary Drug Delivery Systems

Although many advances have been made in the use of AI in the delivery of PBDs through inhaler systems, many clinical, regulatory, technological, and socioeconomic limitations affect the widespread use of smart inhaler drug and device combination systems. Although long-term patient experiences with digital inhalers have been reported, there is variability in the engagement of long-term patients, especially older patients and those with lower digital skills. Ongoing engagement patient is critical for providing continuous high-quality real-world data to refine and validate AI models. Variability in long-term patient engagement creates a major barrier to realizing the multi-scale predictive and personalized modeling of smart inhalers [57,58,64,65].

According to a Cochrane systematic review of digital inhalers in asthma and chronic obstructive pulmonary disease (COPD), although digital inhalers may improve patient adherence, inhaler multi-scale studies continue to provide limited evidence with respect to sustained clinical benefits and long-term patient engagement because of differences in study design, patient adherence, and technical performance [58]. Additionally, the ACCEPTANCE study protocol revealed that in the global asthma population, the median percentage of patients adherent to medication ranged from 13% to 52%, suggesting that continuous education and behavioral and technological support are essential for sustaining patient engagement while patients use electronic monitoring devices [59]. The study also revealed that the implementation of smart inhalers over 12 months would require ongoing patient follow-up, the maintenance of device connectivity, and patient education to promote continued engagement [59].

However, there are substantial technical limitations to this approach. Studies evaluating the performance of inhaler monitoring devices have indicated that sensor-based inhaler systems are subject to synchronization problems, battery-related interruptions, and variability in inhaler actuation recordings over extended periods [60]. Electronic monitoring systems also require a compatible smartphone, internet connectivity, and stable cloud-based infrastructure, which may decrease their usability in older or rural populations [60,64,65]. In protocol-driven smart inhaler trials, such as ACCEPTANCE and OUTERSPACE, researchers intentionally included extra support from patient assistance systems and replacement monitoring devices to reduce the chances of data loss and device malfunction during extended follow-up periods [59,61].

The FDA’s decision to classify AI-assisted inhalers and other platforms using AI as Software as a Medical Device (SaMD) has created regulatory challenges related to the continuous validation of adaptive AI algorithms, development of effective cybersecurity structures against increasingly sophisticated threats, and operationalization of algorithm transparency associated with “black-box” models. These areas of concern require new regulatory frameworks and investment from developers. As AI models have primarily been trained using digitally connected populations, they may perform poorly in older or socioeconomically disadvantaged patient populations, leading to potentially greater disparities in global healthcare for respiratory issues [58,64]. The financial costs associated with smart inhalers are prohibitive for some patients. In the case of smart inhalers, these costs include hardware (i.e., the smart inhaler device), software (i.e., mobile app), ongoing service contracts (e.g., cloud storage), and the increased time needed to monitor patients by healthcare providers [59,64,65]. Real-world implementation studies have highlighted key factors contributing to the successful implementation of AI-enabled inhalation systems, including technical optimization, cost-efficient infrastructure, user-centered design, equitable access to digital technologies, and long-term clinical validation.

Furthermore, several AI systems mentioned in the available publications were created with the help of relatively narrow datasets; thus, they are not sufficiently robust and cannot be applied to various patient categories, inhalers, and manufacturing contexts [1,50,58,64]. Therefore, future research should emphasize the need for multicenter validation, external validation with the help of independent datasets, the standardization of reporting practices, and the explainability of AI for better clinical reliability and regulatory approval [48,50,66].

## 8. Future Perspectives

Advancements in AI are expected to transform pulmonary drug delivery, resulting in improved therapeutic outcomes and shaping the future of precision medicines. Next-generation smart inhaler platforms can be integrated in a multimodal way, combining data from the use of smart inhalers with information from wearable sensors, home spirometry tests, tele-rehabilitation technologies, or even the Internet of Things for real-time monitoring, enabling a comprehensive approach to respiratory health [67]. Another promising development is the integration of digital twin technology with cyber–physical systems and respiratory medicine simulations. Such an approach could help in better understanding chronic diseases and provide personalized and timely interventions for patients [55]. The integration of generative AI (GenAI) and explainable AI (XAI) improves clinical decision-making processes, workflow optimization, and health outcomes while addressing important concerns related to limited data, bias, and patient privacy [66]. There is also a need to construct transparent and trustworthy predictive models within the realm of data science and AI to enable closed-loop management in terms of data collection and individualized intervention. It contributes significantly to effective home-based management without exacerbations or hospitalizations [68]. Although pulmonary drug delivery systems powered by AI have shown promising outcomes, several AI models currently in use remain in the proof-of-concept stage or in the early stages of validation [1,50]. Future studies should focus on the large-scale validation of these models. In addition, it is important to have an appropriate evaluation framework for AI models so that their performance can be understood and interpreted [48,50,66]. Despite these promising developments, the future of AI-based pulmonary drug delivery technologies must involve collaborative efforts from experts in pharmaceutical sciences, medicine, regulation, and data science to construct robust legal frameworks, address privacy concerns, and ensure that AI is used effectively.

## 9. Conclusions

Technological advancements in pulmonary drug delivery, coupled with AI and ML, have significantly affected personalized and precision therapies. AI and ML improve the predication of formulation parameters, manufacturing and process variables, drug–device compatibility, optimization parameters, user-interface properties, patient adherence, and compliance. Artificial neural networks, deep learning algorithms, random forests, computational modeling, and AI-based QbD predict drug–device parameters such as MMAD, FPF, emitted dose, and regional lung deposition without the need for extensive and rapid experimental approaches. In addition, AI-based smart inhaler devices coupled with digital sensor cloud computing technologies and real-time monitoring systems support manufacturing process optimization and improved treatment adherence. Furthermore, AI-based digital twin systems optimize the process reproducibility, reliability, and scalability of pulmonary drug–device combination products.

## Figures and Tables

**Table 1:** Comparison of AI methods used in pulmonary drug delivery.

AI Method	Typical Dataset	Performance Metrics	Advantages	Limitations	Pulmonary Drug Delivery Application
Artificial Neural Networks (ANNs)	Formulation variables, aerosol performance datasets	R^2^, RMSE	Excellent nonlinear prediction capability	Requires large datasets, limited interpretability (“black-box”)	Prediction of MMAD, FPF, emitted dose and formulation optimization [1,46]
Random Forest (RF)	Formulation and process datasets	Accuracy, feature importance	Robust against overfitting, identifies important variables	Moderate computational complexity	Formulation optimization, QbD, feature selection [1,47,48]
Support Vector Machine (SVM)	Small-to-medium formulation datasets	Accuracy, RMSE	Performs well with limited datasets	Limited scalability for large datasets	Aerosol performance prediction and optimization [1,48]
Deep Learning (CNN/MLP)	SEM images, inhaler sensor data, imaging datasets	Accuracy, R^2^	Learns complex image and sensor features	Requires large annotated datasets, poor interpretability	Particle morphology analysis, smart inhalers, imaging applications [17,47]
CFD-ML / Digital Twin Models	CFD simulations and process data	Prediction error, MMAD shift	Supports personalized aerosol delivery and manufacturing optimization	Computationally intensive, requires external validation	Aerosol deposition prediction, scale-up and process optimization [16,49,50]

**Table 2:** Applications of AI in pulmonary drug delivery.

AI Application	Function	Tools	Outcome
Formulation optimization	Predict excipient ratios and particle properties	RF, SVM, Gradient Boosting, AI-assisted QbD	Improved FPF and emitted dose [46,47,50]
CFD-assisted modeling	Simulate aerosol transport and deposition	CFD, AI-based mechanistic models, Digital twin systems	Enhanced lung targeting [16,52,53,54,55]
Smart inhalers	Monitor inhalation technique and adherence	RS01X, flow sensors, ML algorithms	Improved patient compliance [17,18,19,56,57,58,59,60,61]
AI–QbD systems	Identify CPPs and CQAs	AI-assisted QbD frameworks, ML models	Reduced experimental workload [35,48,49]
Imaging analysis	Evaluate nanoparticle distribution	LungVis 1.0, 3D imaging systems	Better understanding of lung retention [62]

## Data Availability

No new data were created or analyzed in this study. Data sharing is not applicable to this article.
